# Development and Antibacterial Activity of Cashew Gum-Based Silver Nanoparticles

**DOI:** 10.3390/ijms14034969

**Published:** 2013-03-01

**Authors:** Patrick V. Quelemes, Felipe B. Araruna, Bruna E. F. de Faria, Selma A. S. Kuckelhaus, Durcilene A. da Silva, Ronaldo Z. Mendonça, Carla Eiras, Maria José dos S. Soares, José Roberto S. A. Leite

**Affiliations:** 1 Biotec—Biodiversity and Biotechnology Research Center, Federal University of Piauí, Parnaíba, PI 64202020, Brazil; E-Mails: pquelemes@gmail.com (P.V.Q.); araruna@ufpi.edu.br (F.B.A.); durcileneas@yahoo.com.br (D.A.S.); carla.eiras.ufpi@gmail.com (C.E.); 2 Medical Plants Research Center, Federal University of Piauí, Teresina, PI 64049550, Brazil; 3 Federal Institute of Education, Science and Technology of Piauí, Parnaíba, PI 64210260, Brazil; 4 Laboratory of Forensic Ridgeology, Institute of Civil Identification of Police, II/PCDF, 70610200 DF, Brazil; E-Mail: bruna.unb@hotmail.com; 5 Laboratory of Cellular Immunology, School of Medicine, University of Brasília, Brasília, DF 70910900, Brazil; E-Mail: selmask@gmail.com; 6 Laboratory of Parasitology, Butantan Institute, 05504900, São Paulo, SP, Brazil; E-Mail: zucatelli@butantan.gov.br; 7 Department of Veterinary Morphophysiology, Federal University of Piauí, Teresina, PI 64049550, Brazil; E-Mail: mrsapijf@gmail.com

**Keywords:** silver nanoparticles, cashew gum, antibacterial activity, cytotoxicity

## Abstract

The present study describes the development of a green synthesis of silver nanoparticles reduced and stabilized by exuded gum from *Anacardium occidentale* L. and evaluates *in vitro* their antibacterial and cytotoxic activities. Characterization of cashew gum-based silver nanoparticles (AgNPs) was carried out based on UV–Vis spectroscopy, transmission electron microscopy and dynamic light scattering analysis which revealed that the synthesized silver nanoparticles were spherical in shape, measuring about 4 nm in size with a uniform dispersal. AgNPs presented antibacterial activity, especially against Gram-negative bacteria, in concentrations where no significant cytotoxicity was observed.

## 1. Introduction

The ability of pathogenic bacteria to resist antibacterial agents has evolved since the start of antimicrobial therapy and it still constitutes a serious problem in clinical practice; limiting the arsenal of drugs available and requiring the development of new substances to combat them [1–4]. In this context, inorganic agents have been considered excellent candidates for research because of their high antibacterial potential [5,6]. Among them, silver compounds and their derivatives are one of the most extensively studied, and a current focus of research involving this metal is the synthesis and evaluation of their antimicrobial activity in the form of nanoparticles [7–11].

Silver nanoparticles have been produced by different routes of synthesis. However, some of them appear to be unconcerned with environmental issues regarding the potential deleterious effect of some of their constituents, while at the same time presenting high levels of toxicity; this requires the development of nanoparticles using biocompatible and biodegradable materials, which is the purpose of “Green synthesis” [12–14]. Some of the reducing and/or stabilizing agents used for the production of silver nanoparticles by green synthesis that stand out are: microorganisms like bacteria and fungi [15–17], chitosan [18,19]; vegetable extracts [20,21]; seaweed [11,22] and natural gums [10].

Cashew gum is a natural product extracted with low cost and is an easily available source, the *Anacardium occidentale* L. tree, widely present in the northeastern part of Brazil. It is a non-toxic, complex heteropolysaccharide exuded either naturally or by making incisions in the trunk or the branches of the trees; it is seen as a yellowish resin. It is reported to contain: b-d-galactose (72%), a-d-glucose (14%), arabinose (4.6%), rhamnose (3.2%) and glucuronic acid (4.7%) [23].

In this study the development of cashew gum-based silver nanoparticles (AgNPs), reduced and stabilized by gum exuded from *Anacardium occidentale* L., a natural, renewable and sustainable source, is presented. The cashew gum-based silver nanoparticles (AgNPs) were characterized by UV–Vis spectroscopy, Transmission Electron Microscopy (TEM) and Dynamic Light Scattering (DLS) analysis followed by evaluation of their antibacterial and cytotoxic activities.

## 2. Results and Discussion

**Figure 1:**
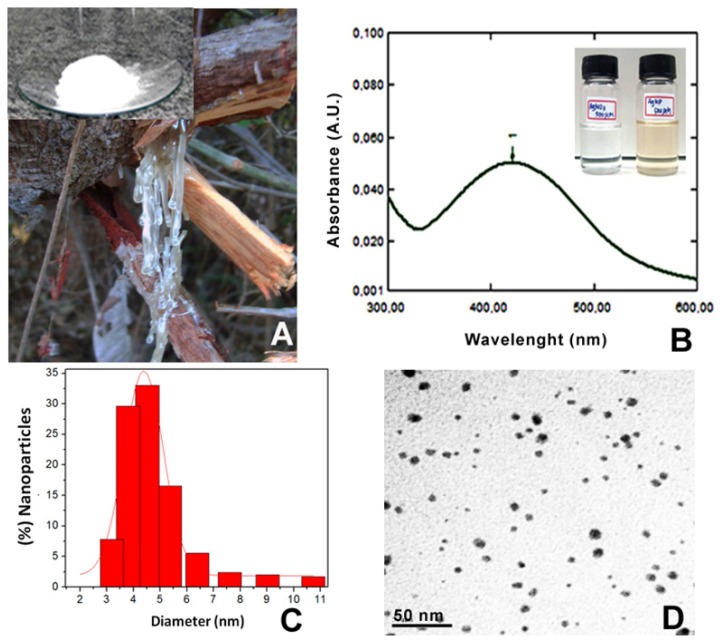
(**A**) Gum exuded from the stem of the cashew (Inset: cashew gum purified); **(B**) UV-Vis absorption spectrum of the cashew gum-based AgNPs; the arrow indicates the maximum absorbance at 420 nm (Inset: Photography comparing the color of the solutions of AgNO_3_ and AgNPs); (**C**) Histogram showing the particle size distribution (4.38 ± 0.07 nm) measured by Dynamic Light Scattering (DLS); (**D**) Transmission Electron Microscopy (TEM) image of cashew gum-based AgNPs (scale bar 50 nm).

Silver nanoparticles were formed by the reduction of Ag^+^ into Ag^0^ with the addition of cashew gum (0.3%) to a solution of 1 mM of AgNO_3_. The colorless solution of AgNO_3_ turned yellowish in color, indicating the formation of silver nanoparticles. The formation of silver nanoparticles was monitored by UV–Vis absorption spectra at 300–600 nm where a band was detected at 420 nm. The silver nanoparticle size, morphology and distribution were analyzed by TEM and DLS (Figure 1).

In this paper, we show that the heteropolysaccharide isolated from the cashew gum acts as a reducing and stabilizing agent in the synthesis of AgNPs. Despite the strong stabilizing potential of cashew gum, the apparent low yield in the conversion Ag^+^ into Ag° can be correlated to a low potential reducing this natural product when using this new green synthesis route. However, the synthesis shown has the characteristic of being fast and low cost. Previous studies have shown that natural gums may have a greater reducing potential, however through routes that include steps such as use of autoclave [10] that can alter the characteristics of natural products; or routes involving reactions with very long durations [24].

Nanoparticle preparation with biopolymers has several advantages over conventional synthetic chemical agents such as the presence of macromolecular chains of these biopolymers that posses a large number of hydroxyl groups that can complex well with the metal ion [4]. The natural polysaccharides may also possess negatively charged carboxyl groups, which can electrostatically interact with Ag^+^ ions to form a complex as the part of Ag nanostructure [25].

We selected, cataloged and standardized bacterial strains with significant clinical importance in this study. These microorganisms are responsible for numerous diseases, cases of hospital infection, colonization of medical devices, and have the ability to acquire resistance [26]. Furthermore, they are strains commonly used in studies of antibacterial activity of AgNPs [10,16,19].

The MIC of an antibacterial agent for a given organism is the lowest concentration required to inhibit the growth of a bacterial inoculum in a standard test. The MBC is the minimal concentration of antibiotic that kills the inoculum and can be determined from broth dilution MIC tests by subculturing to agar media without antibiotics. An agent is usually regarded as bactericidal if the MBC is no more than four times the MIC [27]. In relation to the MICs observed for the Gram-positive bacteria, there was a greater susceptibility to *S. epidermidis*. For this group of bacteria, there was no difference in MIC between AgNPs and AgNO_3_ (Table 1). For the Gram-negatives, AgNPs presented a MIC of 6.75 μgAg/mL for *E. coli* ATCC 35218, half the value obtained with AgNO_3_ (13.5 μgAg/mL). AgNPs also presented a MIC of 3.37 μgAg/mL for *P. aeruginosa* (the lowest observed in the Gram-negative bacteria), it also presenting a higher potency compared to AgNO_3_ (MIC of 6.75 μgAg/mL) (Table 1). In relation to MBC test, *E. coli* ATCC 35218 again showed a higher susceptibility to AgNPs (CBM of 6.75 μgAg/mL) than to AgNO_3_ (CBM of13.5 μgAg/mL) and it showed identical results to the MIC test for both agents (Table 1).

The higher susceptibility of Gram-negative bacteria to AgNPs may be due to the rapid internalization of the nanoparticles through their thin cell walls, which are low in peptidoglycan, and thus inactivating and/or altering the protein structure, leading to cell death. Due to Gram-positive bacteria possessing a cell wall containing a thick layer of peptidoglycan, it becomes more difficult to absorb AgNPs in the cytoplasm, which might explain the need for a higher concentration of agent being required for antibacterial action [22].

In all tested bacteria, there was no antibacterial activity of cashew gum, whose concentrations were tested at dilutions concomitant of their concentration in the solution of AgNPs produced. The MICs of antibiotics patterns presented in accordance with standards established by the Clinical Laboratory Standards Institute [28] (Table 1).

**Figure 2:**
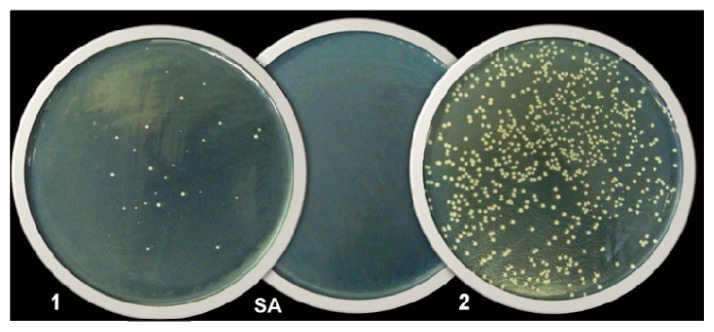
Example in which the antibacterial activity of AgNPs was greater than AgNO_3_. Effect of 13.5 μgAg/mL of (**1**) AgNPs and (**2**) AgNO_3_ on *Staphylococcus aureus* ATCC 29213 observed in the CBM test. (**SA**) Sterile Agar.

**Figure 3:**
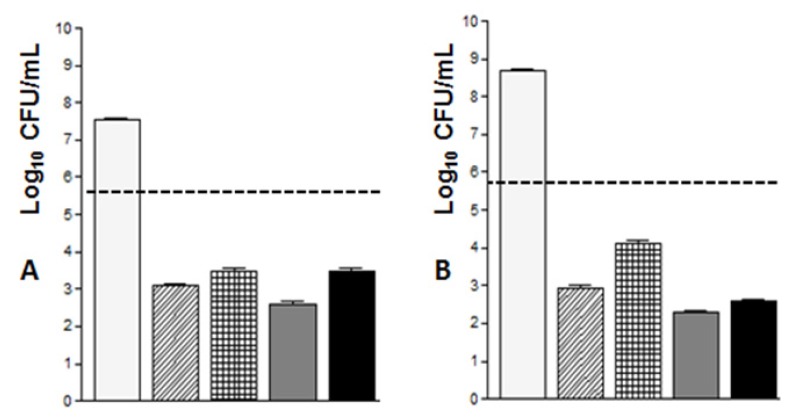

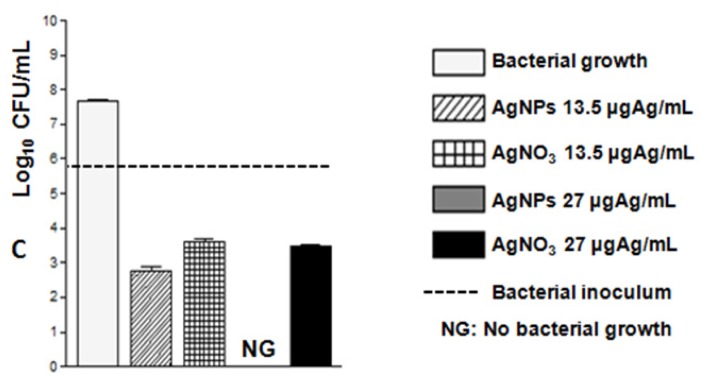
Antibacterial effect of 13.5 and 27 μgAg/mL of AgNPs and AgNO_3_ on: **(A**) *Staphylococcus epidermidis*; (**B**) *Staphylococcus aureus* ATCC 29123 and (**C**) *Enterococcus faecalis*, in an exposure time of 24 h. Each column represents the mean ± SEM of CFU/mL.

The result of the determination of minimum bactericidal concentration for *S. aureus* ATCC 29212 and *S. epidermidis* was greater than 27 μgAg/mL (the highest concentration tested) (Table 1). However, a smaller number of colonies resulting from the effect of AgNPs were observed when compared to the results obtained by AgNO_3_ for all tested concentrations (Figure 2). In this case, in all Gram-positives bacteria, AgNPs caused a reduction higher than or equal to 3 Log_10_ CFU/mL in relation to the inoculum (Figure 3), which also characterizes bactericidal activity [27].

Tran *et al*. [19] cited as to the possible action mechanisms of AgNPs: Ag^+^ ions are supposed to bind to sulfhydryl groups, which lead to protein denaturation by reducing disulfide bonds; Ag^+^ can complex with electron donor groups containing sulfur, oxygen, or nitrogen that are normally present as thiols or phosphates on amino acids and nucleic acids; AgNPs have been found to attach to the surface of the cell membrane and disrupt its function, penetrate bacteria, and release Ag^+^; AgNPs target the bacterial membrane, leading to a dissipation of the proton motive force.

According to Chatterje *et al*. [29], among the different nanomaterials with antibacterial properties, metal nanoparticles are the most potent and promising agents. However, to use them commercially, detailed investigations on the mechanism of microbial death and toxicity in mammalian cells must be performed. In this study, we performed a preliminary study of the cytotoxicity of AgNPs in order to investigate its effect on VERO cells, recommended for studies of cytotoxicity of biomaterials by international norms [30].

**Figure 4:**
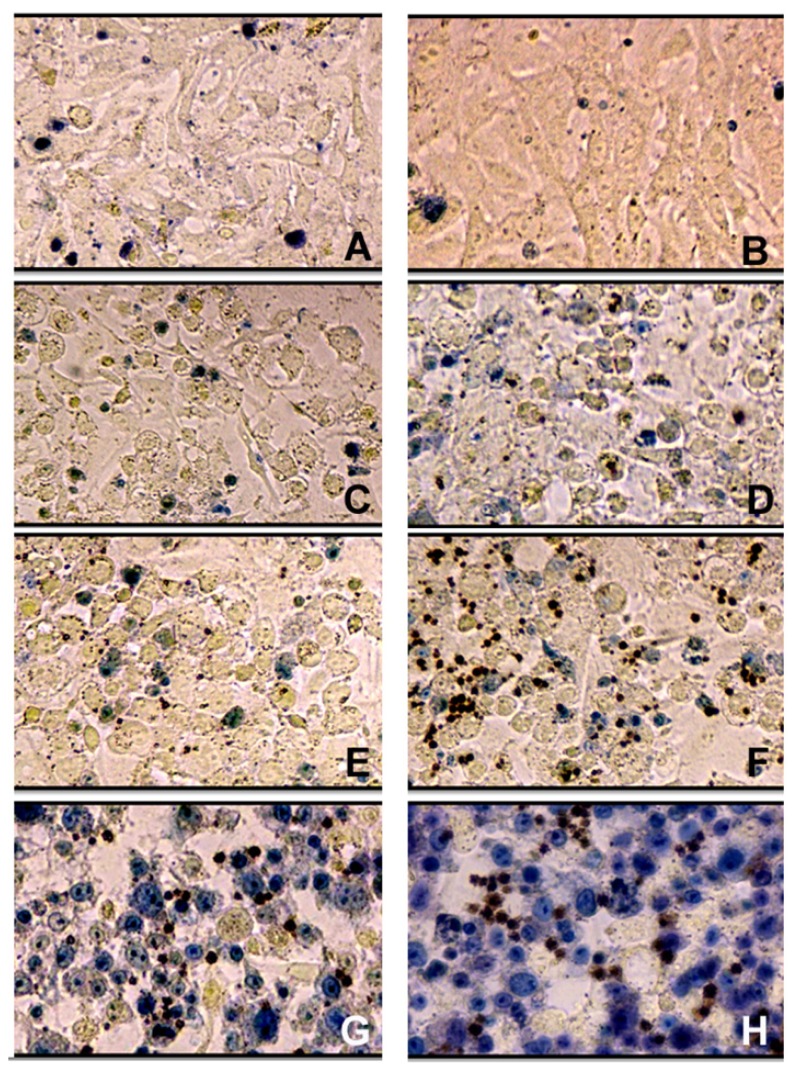
Morphological evaluation of VERO cells in staining with trypan blue after 48 hours of incubation, exposed to the action of the agents analyzed: (**A**) growth control; (**B**) Cashew gum-750 μg/mL; (**C**) AgNPs-6.75 μgAg/mL; (**D**) AgNO_3_-6.75 μgAg/mL; **(E**) AgNPs-13.5 μgAg/mL; (**F**) AgNO_3_-13.5 μgAg/mL; (**G**) AgNPs-27 μgAg/mL; **(H**) AgNO_3_-27 μgAg/mL.

Initially a qualitative assessment of the effect of agents on the viability of cell culture through a morphological analysis of cells using trypan blue dye was performed, in which cells with damages in membrane were stained. Figure 4 shows photomicrographs obtained from the standard view of the same field focal and localized region in the center wells of the plate. Cashew gum, at the highest concentration used (750 μg/mL), did not change the morphology of the cells in comparison to the one observed in the control group. However, solutions of AgNPs and AgNO_3_ caused an increase in the number of damaged cells as a result of higher concentrations. Furthermore, in the cell culture wells subjected to the action of these agents, the appearance of colored blackish circular structures was observed, especially in higher concentrations, with seemingly larger amounts in cells treated with AgNO_3_. It is likely that these structures result from the formation of silver crystals reduced due to possible reducing agents present in the cell culture medium such as carbohydrates and proteins (Figure 4).

**Figure 5:**
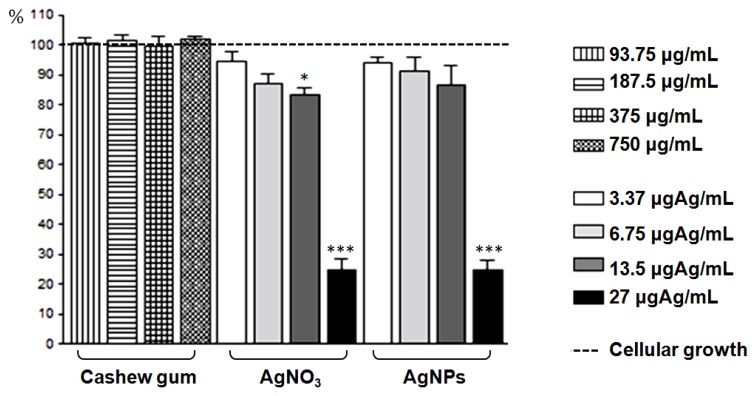
Effect of cashew gum, AgNO_3_ e AgNPs on VERO cell adherence, after 48 hours of exposure. Each column represents the mean ± SEM * *p* < 0.05, *** *p* < 0.001 compared to the control group of cell growth (One-way analysis of variance followed of Dunnett’s multiple comparison test using GraphPad Prism 4.0).

Figure 5 shows the results of the quantitative assessment of cytotoxicity of the tested agents. It was found that there was no reduction in the number of adhered cells in the group treated with cashew gum. The AgNPs action did not cause statistically significant decrease in the percentage of adhered cells from 3.37 to 13.5 μgAg/mL. However, the highest concentration of AgNPs and AgNO_3_ (27 μgAg/mL) resulted in a cell adherence of about 25%, which is the concentration that presented significant cytotoxicity to VERO cells.

It was found, however, that the minimum inhibitory concentrations of all bacteria tested are in a concentration range of AgNPs (between 3.37 and 13.5 μgAg/mL) in which there was no observed significant cytotoxic activity compared to the control. This fact is more significant in respect to Gram-negative bacteria, for which the bactericidal concentration values are also described in the same range.

This work focused primarily in the development of a new green synthesis route and application of this nanomaterial as an antibacterial solution. We performed some preliminary tests of cytotoxicity in cultured mammalian cells. However, subsequent studies related to the cytotoxicity of these cashew gum-based silver nanoparticles should be performed, such as checking its influence on cellular metabolism and its genotoxicity on VERO cells as well in other cell lines.

The AgNPs presented in this study were carried out by means of a fast and low cost “Green synthesis” using an accessible, sustainable and renewable natural product. It is too early to speculate how the formation of cashew gum-based AgNPs may be used in the prevention or in the control of bacterial infections. However the amount of biotechnological products containing AgNPs in its composition is increasing every day. For example it is being used in impregnation of wound dressings, medical devices, dental materials, fabrics, among others [8,31,32], besides the combination with antibiotics for the observation of a synergistic antibacterial effect [16,17]. Further studies may be conducted with varied physicochemical parameters to improve the efficiency of the AgNPs reaction synthesis with cashew gum, which could increase the antibacterial activity and reduce the cytotoxic effects.

## 3. Experimental Section

### 3.1. Cashew Gum Purification

The cashew gum purification was performed following the method of Silva [33] with some modifications. The gum collected from *Anacardium occidentale* L. was solubilized in ultrapure-water at room temperature in a concentration of 5% *w*/*v*, under magnetic stirring for 12 h. After its filtration to remove impurities, the pH of the solution was adjusted to 7 and then NaCl P.A., in proportion to the gum, was added to obtain the polymer as a sodium salt. After stirring it, for the solubilization of the salt, the gum was precipitated with ethanol P.A. at the proportion of 1:4 *v*/*v* (solution of gum:ethanol), vacuum filtered and dried. Then the product was dissolved again in a concentration of 5% *w*/*v* and subjected two more times to the same procedure described above, except for the addition of NaCl, which has its amount halved for the first repetition and then reduced to zero.

### 3.2. Synthesis and Characterization of Silver Nanoparticles

Solutions of silver nitrate (AgNO_3_) 1 mM and cashew gum (0.3% *w*/*v*), previously solubilized under stirring for 12 hours were prepared. The synthesis was performed in an open glass reactor with magnetic stirring, in temperature-controlled water bath at 78 ± 2 °C by mixing the two solutions (1:1 *v*/*v*) for 60 min. The yellowish color of the silver nanoparticles was observed by digital camera and the synthesis of silver nanoparticles was monitored by UV-Vis-NIR scanning spectrophotometer (UV-3101 PC, Shimadzu, Japan) over the range of 300–600 nm. For the Transmission Electron Microscopy (TEM), a drop of colloidal solution consisting of silver nanoparticles was dispensed directly onto a carbon-coated copper grid and allowed to dry completely in a vacuum desiccator. The images were obtained using a JEOL transmission electron microscope (Model JEM 2100, Tokyo, Japan) equipped with an EDX attachment at 200 kV. The hydrodynamic diameter was measured by dynamic light scattering with laser with a wavelength of 633 nm and afixed scattering angle of 90°. Particle size was measured considering the particle as being spherical. Each sample was measured three times so as to have three replicate samples.

### 3.3. Evaluation of Antibacterial Activity of AgNPs

To study the antibacterial properties of AgNPs, four strains of Gram-positive bacteria were selected: *Staphylococcus aureus* ATCC 29213; Methicillin-Resistant *S. aureus* COL (MRSA) [34]; *Staphylococcus epidermidis* ATCC 12228 and *Enterococcus faecalis* ATCC 29212; and four Gram-negatives were also selected: *Escherichia coli* ATCC 25922; *Escherichia coli* ATCC 35218; *Pseudomonas aeruginosa* ATCC 27853 and *Klebsiella pneumoniae* ATCC 700603. The microorganisms were cultured in Mueller-Hinton agar at 37 °C for 24 hours in aerobic conditions. Then a suspension of bacterial strains with an optical density of McFarland of 0.5 (1 × 10^8^ CFU/mL) was made in an isotonic sodium chloride 0.85% solution. Later in time, this solution was diluted ten times (1 × 10^7^ CFU/mL) and used as inoculum in the experiments described below.

#### Determination of the Minimum Inhibitory Concentration (MIC) and Minimum Bactericidal Concentration (MBC) of AgNPs

MIC was determined according to CLSI [28] with some adaptations using 96-well microdilution plate where the strains (concentration of 5 × 10^5^ CFU/mL) were exposed to two-fold dilution series of the AgNPs ranging from 27 to 0.42 μgAg/mL. The same procedure was used to determine the MIC of the following controls: cashew gum, AgNO_3_, and standard antibiotics effective against the bacterial strains tested. At the end of the microdilution, concentrations ranged from 27 to 0.42 μgAg/mL for AgNO_3_; 750 to 5.85 μg/mL for cashew gum and 32 to 0.25 μg/mL for antibiotics. Sterile Mueller-Hinton broth was used as the negative control and inoculated broth was used as the positive control. MIC was defined as the lowest concentration of agent that restricted growth to a level lower than 0.05 at 600 nm (no visible growth).

For the MBC determination, aliquots (50 μL) from all wells with concentrations higher or equal to the MIC concentrations were sub-cultured on Mueller-Hinton agar. MBC was defined as the lowest concentration that enabled no growth on the agar (99.9% kill). If bacterial growth occurred, the units forming colonies (UFC) were counted in order to observe a possible decrease in their number relative to the increased concentration of the agent tested. All assays were performed in triplicate.

### 3.4. Evaluation of Cytotoxicity of AgNPs

Vero mammalian cells (African green monkey kidney fibroblast) ATCC CCL-81 were used in this study. For a short duration, Vero cells maintained in Leibovitz L-15 (VITROCELL, Campinas, Brazil) and supplemented with 10% heat-inactivated calf serum were seeded at a density of 5 × 10^4^ cells per well into 96-well culture microplate. Cells groups treated with different concentrations of AgNPs (ranging from 27 to 3.37 μgAg/mL), AgNO_3_ (27 to 3.37 μgAg/mL) and cashew gum (750 to 93.75 μg/mL), as well as a control group of cell growth, were incubated at 37 °C in a 5% CO_2_ atmosphere. After 48 hours of incubation, trypan blue dye was applied to all wells and after 15 min the content of the wells was gently aspirated and the plate was taken for microscopic observation under 400 × magnification (NIKON TS 100).

In the next step, crystal violet dye was applied to the wells for 10 min, then the content of the wells was removed and the plate was washed with water. After drying at room temperature, the plate was again taken to observation by microscopy observed under 400× magnification and the digital images of the remaining adhered cells were captured using NIS-Elements F software. The captured images were standardized to cover the central area of each well and through them the percent of adhered cells was calculated in relation to the control group. The results were expressed as the mean ± SEM and analysed by one-way analysis of variance followed by Dunnett’s multiple comparison test. Differences between groups and control were considered significant when *p* < 0.05 (GraphPad Prism software 4.0).

## 4. Conclusions

Cashew gum-based silver nanoparticles showed antibacterial activity with greater effect on Gram-negative bacteria. Cytotoxic effect on VERO cells at the highest concentration of AgNPs evaluated was observed, showing, at this concentration, results similar to those generated by AgNO_3_. There was no significant cytotoxicity at the concentrations of AgNPs necessary to cause bactericidal effects on Gram-negative bacteria.

## Figures and Tables

**Table 1:** Minimum inhibitory concentrations (MICs) and minimum bactericidal concentrations of AgNPs (μgAg/mL), AgNO_3_ (μgAg/mL) and standard antibiotics (μg/mL).

Bacterial strains	AgNPs		AgNO_3_		Antibiotic
	
	MIC	MBC	MIC	MBC	MIC
*S. epidermidis*	3.37	>27	3.37	>27	Oxacilin
ATCC 12228					<0.5
*S. aureus*	13.5	>27	13.5	>27	Oxacilin
ATCC 29213					<0.5
*S. aureus* COL	13.5	13.5	13.5	13.5	Vancomycin
MRSA					1
*E. faecalis*	13.5	27	13.5	>27	Vancomycin
ATCC 29212					2
*E. coli*	6.75	6.75	6.75	6.75	Meropenen
ATCC 25922					<0.5
*E. coli*	6.75	6.75	13.5	13.5	Meropenen
ATCC 35218					<0.5
*K. pneumoniae*	6.75	6.75	6.75	6.75	Meropenen
ATCC 700603					<0.5
*P.aeruginosa*	3.37	6.75	6.75	6.75	Meropenen
ATCC 27853					<0.5
